# Injectable hydrogels delivering therapeutic agents for disease treatment and tissue engineering

**DOI:** 10.1186/s40824-018-0138-6

**Published:** 2018-09-26

**Authors:** Jin Hyun Lee

**Affiliations:** 1 Polymer Technology Institute, Sungkyunkwan University, Suwon, Gyeonggi-Do 16419 Republic of Korea; 2 Department of Polymer Science & Engineering, Inha University, Incheon, 22212 Republic of Korea

**Keywords:** Injectable hydrogels, Therapeutic agent delivery, Crosslinking reaction, Disease and cancer therapy, Tissue repair and regeneration

## Abstract

**Background:**

Injectable hydrogels have been extensively researched for the use as scaffolds or as carriers of therapeutic agents such as drugs, cells, proteins, and bioactive molecules in the treatment of diseases and cancers and the repair and regeneration of tissues. It is because they have the injectability with minimal invasiveness and usability for irregularly shaped sites, in addition to typical advantages of conventional hydrogels such as biocompatibility, permeability to oxygen and nutrient, properties similar to the characteristics of the native extracellular matrix, and porous structure allowing therapeutic agents to be loaded.

**Main body:**

In this article, recent studies of injectable hydrogel systems applicable for therapeutic agent delivery, disease/cancer therapy, and tissue engineering have reviewed in terms of the various factors physically and chemically contributing to sol-gel transition via which gels have been formed. The various factors are as follows: several different non-covalent interactions resulting in physical crosslinking (the electrostatic interactions (e.g., the ionic and hydrogen bonds), hydrophobic interactions, π-interactions, and van der Waals forces), in-situ chemical reactions inducing chemical crosslinking (the Diels Alder click reactions, Michael reactions, Schiff base reactions, or enzyme-or photo-mediated reactions), and external stimuli (temperatures, pHs, lights, electric/magnetic fields, ultrasounds, or biomolecular species (e.g., enzyme)). Finally, their applications with accompanying therapeutic agents and notable properties used were reviewed as well.

**Conclusion:**

Injectable hydrogels, of which network morphology and properties could be tuned, have shown to control the load and release of therapeutic agents, consequently producing significant therapeutic efficacy. Accordingly, they are believed to be successful and promising biomaterials as scaffolds and carriers of therapeutic agents for disease and cancer therapy and tissue engineering.

## Background

The steady growth in the need for the effective treatment of diseases and repair of injured tissues has led to the extensive development of the biomaterials with desirable and enhanced properties. Hydrogels have been noticed as the desirable biomaterials that meet the requirements such as biocompatibility, biofunctionality, and tunable properties for therapeutic agent delivery and tissue engineering [1–4]. They are the materials that compose of hydrophilic polymer network capable of retaining a significant amount of water and remain insoluble in water or biological fluids due to their crosslinking structure. Based on the nature of material, mechanism of gel formation, nature of side group, biodegradability, and physicochemical properties such as the degree of swelling and porosity, they have been classified into synthetic, natural or hybrid hydrogels [5], chemically or physically crosslinked hydrogels [6], cationic, anionic or neutral hydrogels [7], non-degradable or degradable hydrogels [8], low, high swelling or superabsorbent hydrogels [9], and micro-, macro- or superporous hydrogels [10], respectively. Depending on the compositions and synthesis conditions of hydrogels, their properties and structures are determined. Notably, their porous structures, formed due to the three-dimensional network of hydrogels, permit therapeutic agents to be well adhered and entrapped in hydrogels. Also, the kinetics of therapeutic agent release from hydrogels can be controlled by regulating their porous structure in addition to components. Consequently, the studies on the release kinetics of the agents and the structure/components of hydrogels for high loading and efficacy of the agents incorporated in hydrogels have been reported [11, 12]. Accordingly, the hydrogel systems have been considered as adequate scaffolds or carriers of therapeutic agents. Besides, due to their advantages such as biocompatibility, permeability to oxygen and nutrient, physical properties similar to the characteristics of the native extracellular matrix (ECM), and tunable physical and mechanical properties, they have been vigorously researched for various biomedical applications [13–15]. However, the implantation of pre-formed hydrogels at a desired site in the body demands an invasive surgical procedure that can cause the patient’s pain and discomfort as well as the cost and time. Thus their clinical uses are limited.

**Fig. 1:**
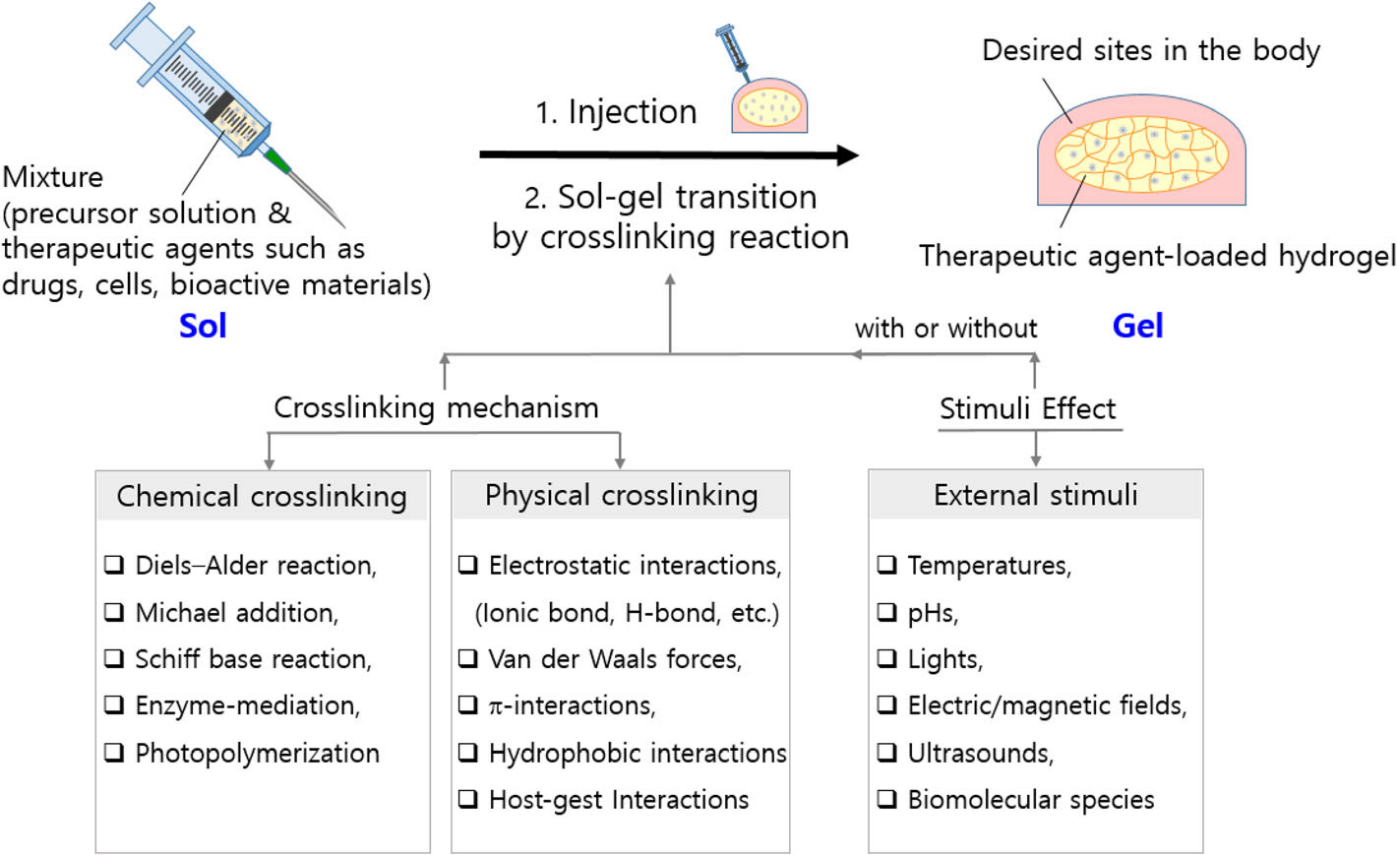
The schematic illustration of the formation procedure of injectable hydrogels containing therapeutic agents through the sol-gel transition induced by physical or chemical crosslinking reactions with or without external stimuli after injection (above). The diagrams of the crosslinking mechanisms (chemical and physical crosslinking) and external stimuli contributing to the sol-gel transition (below)

Injectable hydrogels have the benefits to overcome such drawbacks in the medical use of pre-formed hydrogels. They not only have the typical advantages of conventional hydrogels as mentioned above but can also be injected with minimal invasiveness into target sites and used for irregularly shaped sites. Accordingly, they have been developing as a promising and successful material system for many biomedical applications including the delivery of therapeutic agents such as drugs, cells, and bioactive molecules for the treatment of inflammatory and infectious diseases and cancers and for the repair and regeneration of tissues such as bone, cartilage, muscle, and skin [16]. The schematic illustration of the formation of injectable hydrogels via the sol-gel transition caused by crosslinking and the diagram of crosslinking reaction is shown in Fig. 1. There is a mixture of the polymer/monomer solution (or called precursor) and the therapeutic agents in the syringe (on the left upper side). The mixture is feasibly administrated to a desired site in the body because its viscosity is low enough to be injected through a syringe needle. Then, the therapeutic agent-loaded hydrogel is formed by crosslinking reaction (on the right upper side), where its viscosity drastically increases during the phase transition from sol to gel. The changes in the viscoelastic behavior of injectable hydrogels throughout the phase transition are examined by characterizing their rheological properties [17, 18]. The gelation is typically occurred by forming the crosslinks in the hydrogels via chemically or physically crosslinking reactions. Also, the physical gelation is driven by non-covalent bonds and also often affected by external stimuli. The diagram in Fig. 1 shows the factors contributed to the sol-gel transition as follows: in-situ chemical reactions (Diels-Alder reactions, Michael additions, Schiff base reactions, enzyme-mediations, photopolymerizations), physical interactions (electrostatic interactions including ionic and hydrogen bonds, van der Waals forces, π-interactions, hydrophobic interactions), and external stimuli (temperatures, pHs, lights, electric/magnetic fields, ultrasounds, and enzymes).

Considering factors for designing injectable hydrogels, which are their composition, biocompatibility, biodegradability, chemical and physical properties, and so on, will determine their biological, morphological, physical, and mechanical properties of the solidified hydrogels for the uses. Injectable hydrogels have been prepared by mainly formulating the Food and Drug Administration (FDA) approved or cytocompatible polymers. Natural polymers including alginate, chitosan, collagen, hyaluronic acid (HA), chondroitin sulfate (ChS), dextrin, gelatin, fibrin, peptide, and silk have been selected. Synthetic polymers such as poly(ethylene glycol) (PEG), poly(ethylene oxide) (PEO), poloxamer (Pluronic®) (PEO-PPO-PEO), polyoxamine (Tetronic®) (PEO-PPO), poly(vinyl alcohol) (PVA), poly(lactic-co-glycolic acid) (PLGA), poly(glycolic acid) (PGA), poly(lactic acid) (PLA), polycaprolactone (PCL), poly(L-glutamic acid) (PLga), polyanhydrides, poly(N-isopropylacrylamide) (PNIPAAm), polyaniline ans so on have been also used. As like conventional hydrogels, the preparations of injectable hydrogels have been achieved via either chemical or physical crosslinking methods. Therapeutic agents are typically entrapped in the hydrogel by mixing with precursor solution, and the sterilization process of the mixture is carried out before injection to the body part. Physically crosslinked hydrogels usually provide a friendly environment to cells and bioactive molecules. They generally show a relatively low mechanical strength, and their morphology and properties are easily altered in external stimuli. On the other hand, the chemically crosslinked hydrogels formed by irreversible covalent bonds have shown the relatively high mechanical strength and stability. Also, the crosslinking reaction could be affected by various external stimuli such as temperatures, pHs, lights, ultrasounds, electric/magnetic fields, and biomolecular species (e.g., enzymes).

In this article, a wide variety of injectable hydrogels recently researched for the use as therapeutic agent carriers or scaffolds for the disease and cancer therapy and the tissue repair and regeneration are reviewed, regarding the chemically and physically crosslinking mechanism and various external stimuli. Besides, their properties and applications of hydrogel systems are described as well.

## Development of injectable hydrogels

### Injectable hydrogels formed by physical crosslinking

Physical crosslinking, one of the crosslinking reaction mechanisms used for the fabrication of hydrogels, generally occurs by the following non-covalent interactions: electrostatic interactions between two oppositely charged ions (e.g., ionic bonds, hydrogen bonds, etc.), hydrophobic interactions, π-interactions, and van der Waals forces including dipole-dipole interactions and London dispersion forces. In addition, some injectable hydrogels can also be prepared by physically self-crosslinking occurred via the interactions such as host-guest interactions without any crosslinker or catalyst. Moreover, various external stimuli often affect the gelation caused by the crosslinking. The injectable hydrogels recently developed by various physical crosslinking are reviewed below.

Ishii et al. (2016) [19] synthesized the polyion complex (PIC) micelles consisting of cationic polyamine-poly(ethylene glycol)-polyamine (PMNT-PEG-PMNT) triblock copolymers with nitroxide radicals (reactive oxygen species scavenger) and polyamine groups, the anionic poly(acrylic acid), and incorporated therapeutic proteins (insulin, bovine serum albumin (BSA), glucose oxidase, neutravidin, avidin, or interleukin-12 (IL-12)) under the physiological condition. Then, redox-active injectable gels (RIGs) were prepared for controlled local delivery of therapeutic proteins, by the ionic bonds formed at the temperature about 30 °C, where the viscosity of PIC micelles has increased via sol-gel transition. In addition, the IL-12 entrapped RIGs did not show noticeable initial burst (may cause systemic toxicity and adverse effect) and did the sustained release of protein, resulting in significant tumor growth inhibition. These results suggested their promising uses as protein carriers. For the local delivery to treat Parkinson’s disease, Ren et al. (2017) [20] mixed the synthesized quaternized chitosan (QCS), gelatin (GT), dopamine (DA, a substance able to treat Parkinson’s disease), and metronidazole (MT, an anti-inflammatory substance and antibiotic to treat various parasitic and bacterial infections) in phosphate-buffered saline (PBS) solution, and prepared the MT- and DA-entrapped hydrogels with higher mechanical strength by oxidizing the mixture at 37 °C. The hydrogels were formed not only by the covalent crosslinking between the amino groups of QCS/GT and the quinoid units of DAs/oxidized DA derivatives through Michael reaction or Schiff base reaction but also by physically crosslinking caused by self-assembling the DAs and DA derivatives grafted onto QCS/GT through hydrogen bonding and π-interaction. The mechanical strength of the hydrogels was improved by forming ionic bonds via electrostatic interaction between cationic quaternary ammonium groups of QCS and GT and negatively charged carboxylic groups of DAs and DA derivatives. The hydrogels exhibited nontoxicity and sustained release of DA and MT. In a study by Xing et al. (2016) [21], a shear-thinning and self-healing collagen-Au hybrid hydrogel system was synthesized for photothermal therapy (PTT) by the electrostatic complexation of positively charged collagen proteins and anionic precursor ions [AnCl_4_]^−^ of gold nanoparticles (AuNPs) and by the biomineralization of AuNPs. Their mechanical properties could be controlled by regulating the content of AuNPs used as crosslinkers and as light absorbers. Meso-tetra(N-methyl-4-pyridyl) porphine tetrachloride (TMPyP), a photosensitive model drug, was incorporated in the cytocompatible hybrid hydrogels for photodynamic therapy (PDT). After intratumoral injection of the hybrid hydrogels containing TMPyP to human breast cancer cell line (MCF-7) planted in mice, the treatment of light (*λ* = 635 nm) irradiation was carried out. The tumor growth suppression and tumor regression were observed with high antitumor efficacy. These results showed the potential usability of the collagen-Au hybrid hydrogels in therapeutic agent delivery and cancer therapy.

Zubik et al. (2017) [22] synthesized thermosensitive poly(N-isopropylacrylamide) (PNIPAAm) and cellulose nanocrystal (CNC) hybrid hydrogels by using free-radical polymerization at the temperature ranges from 36 °C to 39 °C. The covalent bonds between PNIPAAm and CNCs as well as the physical bondings between PNIPAAms and CNCs through van der Waals force and hydrogen bonding interaction were involved in the crosslinking reaction. As expected, the mechanical property of the hybrid hydrogels was enhanced with increasing the content of CNC. MTs, antibiotic substances, were incorporated within the PNIPAAm-CNC hydrogels, and their high loading and sustained release were shown. The authors claimed the capability of the hydrogels for uses as drug carriers and wound dressing matrices.

Baral et al. (2014) [23] developed an injectable peptide-based hydrogel formed from 11-aminoundecanoic acid containing tripeptide Boc-AUDA-PhePhe-COOH (AUDA-11-aminoundecanoic acid, Phe-L- Phe-L-phenylalanine) (P1) via both interactions of π-π interactions between neighboring benzyl groups and hydrophobic interactions between aliphatic parts. Vitamin B12 and vancomycin (an antibiotic) entrapped P1 hydrogels with thixotropy at physiological condition showed cytocompatibility and sustained release of drugs. These results showed the hydrogels are potential candidates for drug delivery use. Feng et al. (2018) [24] first researched the crystallization of guanosine-based hydrogel (20-deoxy-20-fluoroguanosine (^F^G_d_) hydrogel with high anti-virus activity). The crystallization induced by the π-π staking of the ring of ^F^G_d_ and the hydrogel formation induced by blocking π-π interaction were affected by adding silver ions. The synthesized ^F^G_d_Ag hydrogels exhibiting excellent mechanical stability more than six months and high antimicrobial activities were proposed as promising antimicrobial materials.

Cinar et al. (2017) [25] fabricated a self-assembled peptide based-hydrogel consisting of peptide amphiphiles (PAs, hydrophobic part: aliphatic alkyl group and hydrophilic part: amino acid) via hydrophobic interactions between aliphatic parts, hydrogen bonds between beta sheets of protein secondary structure, and electrostatic interactions between charged amino acid groups at pH 7.4. Doxorubicin (Dox, an FDA-approved anticancer drug) was entrapped in the amphiphilic hydrogels. The release of Dox from the hydrogels was monitored, and their PA concentration-dependent release behavior was found. The peptide-based hydrogel showing biocompatible and high therapeutic efficacy was thought to have excellent potential use as a local chemotherapeutic system. Payyappilly et al. (2014) [26] synthesized the triblock copolymers composing of poly(ethylene glycol)-poly(e-caprolactone)-poly(ethylene glycol) (PEG-PCL-PEG)(PECE) which were a micellar form in aqueous solution. The PECE micelles changed into a PECE hydrogel at 37 °C via sol-gel transition caused by hydrophobic interactions between PECE micelles. Compared with Pluronic® (PEO-PPO-PEO), PECE showed a relatively lower viscosity (better for injection) and crystalline structure. In vitro tests, the release behavior of insulin entrapped in the PECE hydrogels was shown to follow a Fickian model of first-order, and it was controlled by regulating the concentrations of insulin and polymer and the temperatures. The PECE hydrogels were thought to be promising as therapeutic agent carriers for chemotherapy including diabetic treatment.

A self-healing self-assembled hydrogel has developed by Li et al. (2015) [27] using the triblock poly(L-glutamic acid)-block-poly(ethylene glycol)-block-poly(L-glutamic acid) ((PLga-*b*-PEG-*b*-PLga) modified with cholesterol (Chol) ((PLga-*b*-PEG-*b*-PLga)-*g*-Chol) and the PLga modified with cyclodextrin (*β*-CD) (PLga-*g*-*β*-CD) via host and guest interaction between *β*-CD and Chol. Their viscoelastic behavior and mechanical properties were investigated regarding the ratio of *β*-CD/Chol, and the highest storage modulus (10 kPa) was obtained at 15 wt% of polymer concentration. The hydrogels also showed significant cytocompatibility and self-healing capacity. The potential application of the hydrogels was considered to be tissue engineering. Loebel et al. (2017) [28] fabricated an injectable and printable hydrogel system showing shear-thinning and self-healing behavior for therapeutic agent delivery applications. First, *β*-CD-modified hyaluronic acid (HA-*g-β*-CD) and adamantine-modified HA (HA-*g*-AD) was synthesized. Then, two polymers were mixed to form a gel through host and guest interaction. The hydrogel with host-gest linkages showed low enough viscosity to be printed or injected when shear force was applied to it. In vivo test, the rat endothelial progenitor cells (EPCs)-entrapped hydrogels showed the extended retention time of EPSs, increased vascularization, and reduced scar tissue formation. These results supported the promising uses of the hydrogel for therapeutic agent delivery and tissue engineering. Later, these two polymers were modified with methacrylate groups UV curable, and during the photopolymerization, they produced the secondary covalent bonds that strengthen the mechanical properties. Besides, the thiol-modified fluorophores were conjugated with peptides to monitor the degradation of the hydrogels subcutaneously injected into mice in that work.

The physical crosslinking and the morphology and properties of injectable hydrogels are often influenced and regulated by external conditions such as temperature, pH, electric/magnetic fields, light, biomolecular species (e.g., enzymes), and so on. These stimuli-responsive hydrogels can be promising “smart” biomaterials for tissue engineering and disease therapy.

The studies on temperature-responsive hydrogels are the most frequently reported. Their physical crosslinking is typically formed by undergoing a sol-gel transition at a lower critical solution temperature (LCST). Sim et al. (2015) [29] fabricated a biodegradable anionic conjugate composed of heparin with thiol groups and poly-(*ε*-caprolactone-co-lactide)-b-poly(ethylene glycol)-b-poly(*ε*-caprolactone-co-lactide) (Hep-PCLA-PEG-PCLA) to carry cationic lysozyme as a therapeutic protein model via the Michael addition between the thiol groups in heparin and acrylic groups of PCLA. The lysozymes were well incorporated with the conjugates due to the ionic interaction between charged conjugates and lysozymes. It was examined that the lysozyme-incorporated conjugates were transferred to a gel state through hydrophobic interactions at around body temperature. The hydrogels demonstrated the decreased initial burst and sustained release of lysozyme. In 2017, Pacelli et al. (2017) [30] developed novel nanodiamonds (NDs)-incorporated thermo-sensitive nanocomposite hydrogels consisting of chitosan and gelatin via electrostatic interactions (hydrogen bonds) and van der Waals forces (dipole-dipole interactions) between chitosan/gelatin and NDs. The NDs enhanced the mechanical properties of hydrogels and did not show cytotoxicity and inflammation in vitro. Human vascular endothelial growth factors (VEGFs) were loaded in the NDs-based nanocomposite hydrogels via the interaction between NDs and VEGFs. The sustained release of VEDFs from the VEDFs-entrapped nanocomposite hydrogels and the high proliferation of human umbilical vein endothelial cells (HUVEC) seeded in the hydrogels were shown, which proposed the potential uses of the NDs-based nanocomposite hydrogels as carriers and scaffolds.

The development of pH-sensitive injectable hydrogels has also been extensively proceeding. For the self-regulated release of insulin, Li et al. (2017) [31] fabricated a pH- and glucose-sensitive self-assembling peptide injectable hydrogel loading catalase, glucose oxidase, and insulin. When blood glucose level increased and pH decreased, then insulin was released by disassembling the peptide hydrogel. On the contrary, when blood glucose level decreased and pH increased, the peptide hydrogels reassembled, stopping the release of insulin. The local pH was controlled by the enzymes transforming glucose into gluconic acid. At the low enough pH, the electrostatic repulsion between the charged lysine/ornithine side groups results in unfolding hairpins. Conclusively, these peptide hydrogels, which were shown to regulate well the blood glucose level, were considered as promising biomaterials for diabetic treatment. Ye et al. (2017) [32] also developed a novel pH-sensitive self-healing HA based hydrogel. HA was modified with cytosine (C) and guanosine (G) using hexamethylenediamine (HMDA), and the hydrogels were formed only at pH 6–8 by crosslinking between HA-HMDA-C and HA-HMDA-G polymers via the hydrogen bonds between the modified HAs. As expected, the higher polymer concentration produced stronger mechanical properties and smaller pore size. Besides, the release of protein model drug, BSA, incorporated in the hydrogels was found to be slower for the hydrogels with smaller pore size. Such HA-based hydrogels were thought to be favorable biomaterials for drug or protein delivery and tissue engineering.

In addition to pHs, electric or magnetic fields have been utilized as other external stimuli for the response or formation of hydrogels. In a study by Qu et al. (2018) [33], pH-sensitive and electric field responsive hydrogels were prepared using antibacterial and conductive chitosan-graft-polyaniline (CP) copolymers and oxidized dextrans (OD, crosslinker) via Schiff base reaction for the use as smart drug delivery systems. Amoxicillin and ibuprofen (model drugs) were loaded in CP/OD hydrogels, and the release rate of the model drugs was increased by increasing electric field voltage. The hydrogels showing pH-dependent degradation and morphology, good cytocompatibility, and release regulated by pHs/electric fields were suggested as ideal biomaterials for smart drug delivery. Wu et al. (2018) [34] developed an injectable self-assembling magnetic supramolecular hydrogel (MSH) composing of PEGylated Fe_3_O_4_ NPs and cyclodextrins (CDs) using amphiphilic PEG-phospholipid (DSPE-MPEG 2000). In addition, an injectable magnetic gellan gum hydrogel (MGH) were prepared as well. The sol-gel transition of the hydrogels was occurred by the temperature change induced by alternating current magnetic field (ACMF). The release behaviors of paclitaxel (PTX, attached to the surface of PEGylated Fe_3_O_4_ NPs) and DOX included in the hydrogels were affected by the magnetically induced heat generation. It was also shown that the controlled release of the drugs at 37 °C permitted chemotherapy and the magnetic hyperthermia treatment was possible at 45 °C. These results showed that the hydrogels demonstrating great therapeutic efficacy could be candidate materials avoiding breast cancer recurrence.

Light, the energy source to synthesize organic materials, can also be another triggering factor in the response of hydrogels. In 2015, Ballios et al. [35] presented injectable, biodegradable and light-sensitive hydrogels composing of hyaluronan (HA) boosting cell survival and methylcellulose (MC) helping cell distribution. Their capabilities of cell delivery and cell survival/integration for the functional repair of retina and brain were tested. Both retinal stem cells (RSCs) and neural stem and progenitor cells (NSCs) were delivered by the HAMC hydrogels in retina and brain, respectively, which results in the enhanced cell survival and visual functional repair. The HAMC hydrogels were considered as promising cell carriers for retina and brain tissue therapy. Kharkar et al. (2017) [36] designed and developed novel light- and reducing environment-sensitive hydrogels using aryl-thiol functionalized PEG (PEG-4-MPA, or PEG-4-PD-MPA that is a photodegradable PEG-4-MPA) and maleimide functionalized PEG or heparin (PEG-2-MI or Heparin-MI) through the Michael reaction between thiol groups and maleimide groups. Various proteins including growth factors including fibroblast growth factor (FGF-2), cytokines, and immunomodulatory agents were entrapped via heparin-associated receptor-ligand interaction during the hydrogel formation. The degradable species, alkyl-thiol-based succinimide thioether linkages were yielded from a reversible Michael reaction, and the photolabile o-nitrobenzyl groups were turned to ketone and amide groups under the applied light (UV with long wavelength or visible light) with clinically safe dose (here, 10 mW cm^− 2^ at 365 nm). Via the breaking processes of linkages, which were controlled by external stimuli of light and reducing environment, the various proteins entrapped within the hydrogels were released. The released proteins successfully maintained the bioactivity of primary human aortic adventitial fibroblasts (AF) cells as well as proliferated the AF cells. The new synthesis design of hydrogels gave a high expectation for therapeutic agent delivery. Wu et al. (2018) [37] developed injectable agar-based composite hydrogels including MoS2/Bi2S3-PEG (MBP, a photoabsorbent) and DOX for photothermal and chemotherapeutic effects, respectively, on the treatment of the malignant tumors. The release of DOX from the AMD hydrogels was regulated by the temperatures changed during MBP nanosheets absorbed NIR laser irradiation light (*λ* = 808 nm). The HT29 cells (human colon cancer cells) planted in mice was highly suppressed by the AMD hydrogels with MBP and DOX, namely, by photothermal treatment and chemotherapy.

For the studies on the effects of ultrasounds, Huebsch et al. (2014) [38] reported that by using ultrasounds, an injectable self-healing alginate-based hydrogel with showing controlled release and enhanced chemotherapy was fabricated via ionic interaction between alginates with a high content of guluronic acid and calcium ions (Ca^2+^). Three therapeutic agents that are mitoxantrone (MX, a drug model), stromal cell-derived factor 1α (SDF-1α, a protein model), and plasmid DNA (pDNA) were loaded in the hydrogels. The time and power of ultrasound application to bioactive agent-loaded hydrogels determined the release behavior of three therapeutic agents and the degradation behavior of the hydrogels through the disruption of ionic bonds in the hydrogels. The growth of xenograft tumor MDA-MB-231 cells more decreased for drugs released from the ultrasound-treated hydrogels, compared with non-treated ones. High mice survival was achieved by the combination of both chemotherapies with MX-loaded hydrogels and simultaneous daily treatment of pulsatile ultrasound. Veronick et al. (2018) [39] attempted to repair bone tissues not only by using osteoblast cells released from the fabricated osteoblast cell-embedded collagen hydrogels but also by utilizing Low-intensity pulsed ultrasound (LIPUS) inducing acoustic radiation force. Since the LIPUS-derived force produced the deformation of hydrogels, the stiffness of collagen hydrogels was controlled by the intensity and power of LIPUS. Additionally, the derived force influenced the response of the osteoblast cells entrapped in the deformed scaffold hydrogels. The successful formation of new bone tissues was achieved by utilizing the LIPUS and cell-loaded hydrogels, which was acknowledged from the expression of both cyclooxygenase2 and prostaglandin E2 (PGE2).

Biomolecular species such as enzymes or metabolites also arbitrate the response of hydrogels. Turner et al. (2017) [40] synthesized gelatin hydrogel microparticles (GHMs) at physiological pHs by the crosslinking reaction occurred via electrostatic interaction between anionic gelatin molecules and cationic vascular endothelial growth factors (VEGFs) or cationic bone morphogenetic protein 2 (BMP2) using genipin crosslinkers. The degradation of GHMs was derived by chromatographically purified collagenase (CLSPA collagenase), and the degradation behavior controlled the release behavior of VEGFs and BMP2s, which induce the expression of cells playing an essential role in osteoblast. The enzyme-mediated degradable hydrogel microparticles with sustained release of growth factors could be good candidate systems for tissue repair. Wickremasinghe et al. (2014) [41] also presented the release of therapeutic agents controlled by the hydrogel degradation through enzyme-mediation. First, the liposome particles including growth factor1 (GF1) agents were prepared via self-assembly of phospholipids. Then, the multi-domain peptides (MDPs, here, the sequence K(SL)3RG(SL)3KGRGDS form) nanofiber hydrogel composing of polar terminal residues (lysine) with alternating hydrophilic (serine) and hydrophobic (leucine) residues was formed by the crosslinking reaction occurred via hydrogen bonding and hydrophobic interactions, with incorporating the GF1-entrapped liposomes and embedding GF2 agents within the fiber hydrogels. The GF2 agents released earlier than the GF1 agents entrapped in liposomes. The release of two GFs (epidermal growth factor (EGF) and placental growth factor-1 (PlGF-1)) and monocyte chemoattractant protein-1 (MCP-1, a cytokine) from the MDP hydrogels with the agents was regulated via the enzyme-mediated degradation of hydrogels and the physical gelation by self-assembly. Also, due to the multi-functionality of the MDP hydrogels, the charged or hydrophobic (or hydrophilic) agents could be incorporated. The results supported the MDPs hydrogels as promising systems for tissue regeneration.

### Injectable hydrogels formed by chemical crosslinking

Injectable hydrogels have been prepared not only by physical crosslinking reactions as discussed above, but also by the crosslinking via in-situ chemical reactions such as the Diels Alder click reactions, Michael reactions, Schiff base reactions, or enzyme-or photo mediated reactions. Recent studies on the injectable hydrogel systems fabricated through such chemical reactions without any toxic crosslinker and condition are described in terms of each reaction as follows.

Diels Alder “click” reaction [42], the addition reaction of a conjugated diene to an alkene or alkyne (dienophile) to produce a cyclic ring structure, is simple but produces high yields. The reaction is typically used for obtaining the complex molecular architectures such as crosslinked structure and gets attention in the fabrication of in-situ injectable hydrogels because the reaction simply proceeds without any catalysts such as copper at room temperature (RT). Koshy et al. (2016) [43] reported the preparation of the transparent and covalently crosslinked gelatin hydrogels spontaneously formed with two different type gelatin polymers respectively modified with tetrazine or norbornene pendent groups via the Diels Alder click reaction between the two functional groups. These gelatin-based injectable hydrogels exhibiting cell-compatible and enzymatically degradable properties were thought to be usable as a synthetic ECM and as a platform matrix for cell culture. Bai et al. (2017) [44] prepared a thermosensitive polymer, Pluronic F127-crosslinked ChS (F127@ChS) via click chemistry between F127 with maleimido (F127-AMI) and furfurylamine-grafted ChS (ChS-furan), and the sol-gel transition of F127@ChS gels formed at 37 °C was examined. Then, the dual crosslinked hydrogels were fabricated via the Diels Alder click reaction between F127@ChS hydrogel and PEG with maleimido (PEG-AMI) acted as a crosslinker and used as scaffolds to load bone morphogenetic protein 4 (BMP-4), extracellular signaling molecule, for bone defect repair. In a recent study by Gregoritza et al. (2017) [45], the 4- and 8-armed polyoxamines with maleimide or furyl groups were immediately formed the polyoxamine hydrogels with various ratios of 4- and 8-armed polyoxamines by thermal gelation at 37 °C. The gradual gelation by the Diels Alder reaction between maleimide and furyl groups was additionally involved, finally resulting in the physical and chemical crosslinked hydrogels. Their mechanical properties were tuned by varying the composition ratio between two different polyoxamines. In addition, they showed a high loading dose and controlled release of bevacizumab, a model antibody, and a triphasic release profile with a low burst. The dual gelation technology that could provide the fast gelation rate and high mechanical properties of hydrogels was thought to be promising for the drug delivery and tissue engineering.

Michael addition reaction [46, 47], the conjugate addition of a nucleophile with an electron poor olefin such as unsaturated carbonyl compound, is a thermodynamically controlled reaction that is base-catalyzed or nucleophile-catalyzed and occurs without toxic reagents. Via the reaction, the polymers with linear, hyperbranched, and crosslinked architectures have been synthesized under ambient condition. Notably, for the fabrication of hydrogels with network structure, the Michael addition step polymerization method is frequently utilized by accompanying together with chain growth (e.g., free radical) polymerization. Several types of Michael addition reaction are thiol-Michael reactions, carbon-Michael reactions, aza-Michael reactions and oxa-Michael reactions. Southan et al. (2018) [48] prepared PEG/TMV injectable hydrogels with high storage modulus for the use as scaffolds for tissue engineering. Their moduli were increased by more incorporating tobacco mosaic virus (TMV) nanoparticles. Cysteine-containing TMV mutants (TMV_Cys_s) were chemically added to PEG-DAs through the Michael addition reaction between the thiol group of TMV and one side of acrylic double bonds of PEG diacrylate (PEG-DA) by irradiating UV light. PEG/wildtype TMV (wt-TMV) hydrogels were also prepared via non-covalent bonding for the comparison. It was observed that the storage moduli of PEG/TMV_Cys_s hydrogels were higher than those of PEG/wt-TMV. In 2015, Wei et al. [49] developed novel self-healing hydrogels composing of N-carboxyethyl chitosan (CEC) with amino groups (first prepared via the Michael addition reaction), oxidized sodium alginate (OSA) with amino groups, and adipic acid dihydrazide (ADH, a crosslinker) with aldehyde groups. The gelation via the formation of the dynamic imine and acrylhydrazone bonds through the reaction between amino groups and acrylic acid groups contributed to their self-healing performance. The high cell loading and controlled release were observed as well. Based on the results, PEG/TMV_Cys_s hydrogels were suggested as potential biomaterials for drug and cell delivery.

The Schiff base reaction [50] is also a nucleophilic addition reaction which forms an imine bond from an amine and a carbonyl compound (*e*. *g*., aldehyde or ketone). This reaction has often been utilized for fabricating the chemically crosslinked hydrogels via the reaction between an amine and a carbonyl group in the components. In a study by Cao et al. (2015) [51], the injectable hydrogels consisting of glycol chitosan (GC) and poly(ethylene oxide-co-glycidol)-CHO (poly(EO-co-Gly)-CHO) were prepared under physiological conditions by forming imine bonds via the Schiff base reaction between amino groups of GC and aldehyde groups of (poly(EO-co-Gly)-CHO) acting as a crosslinker. Their mechanical properties and network morphology were controlled by the content of poly(EO-co-Gly)-CHO. The results of the chondrocytes culture in the hydrogels showed to maintain their viability and phenotype after two weeks. Consequently, the chitosan-based hydrogels were considered as promising matrices for cartilage tissue engineering. Chen et al. (2017) [52] prepared an injectable hydrogel composed of oxidized alginate (OAlg) and carboxymethyl chitosan (CMCS), with includinsist the gelatin microspheres (GMs) containing tetracycline hydrochloride (TH). The composite hydrogels were formed by the Schiff-base reaction between aldehyde groups of OAlg and amino groups of CMCS. The composite hydrogels demonstrated antibacterial and biodegradable properties, which proposed their application in bacterial infection therapy. Wu et al. (2016) [53] reported that the injectable hydrogels consisting of PEG and PEG-b-poly (L-lysine) (PPLL) were fabricated without catalysts at RT via Schiff base reaction between the aldehyde groups in PEG and amino groups in PPLL. The hydrogels carried both metformin (ME) and 5-fluorouracil (5FU) for the treatment of colon cancer. The degradation of the hydrogels and the release of 5FU and ME from the hydrogels were controlled by pHs. The use of dual drugs provided high therapeutic efficacy.

Photo-initiated crosslinking [54], mostly activated by UV and LED visible lights, has been used for preparing various hydrogels for therapeutic agent delivery and tissue engineering. It is because of its advantage that the crosslinking can be carried out at physiological temperature with low heat generation. Aregueta-Robles et al. (2018) [55] recently presented that the PVA-tyramine (PVA-Tyr) hydrogels synthesized via the formation of biphenyl bonds between PVA and tyramine under the irradiation of visible light (*λ* = 400–450 nm) at RT. Here, various combinations of two photoinitiators, tris(2,20-bipyridyl) dichlororuthenium(II) hexahydrate (Ru) and sodium persulfate (SPS), were used. Their physical and mechanical properties were shown to be appropriate for cell culture, but they could not support cell adhesion. Thus, PVA-Tyr with sericin and gelatin (PVA-SG) hydrogels were also fabricated by following similar synthesis steps for including Schwann cells. The PVA-SG hydrogels showed good cytocompatibility and proper adhesion and mechanical properties for nerve cell development, suggesting a promising aspect for the use in nerve regeneration. In a study by Skardal et al. (2017) [56], for wound healing purpose, a heparin-conjugated hyaluronic acid (HA-HP) hydrogel containing amniotic fluid-derived stem (AFS) cells was synthesized using 2-hydroxy-40-(2-hydroxyethoxy)22-methylpropiophenone as an initiator under UV (*λ* = 365 nm) irradiation. As described above, the photopolymerization is a popular method to fabricate the hydrogels to be used in biomedical applications because of its simplicity and convenience. However, photoinitiators may cause a local temperature increase, which may cause the damage of local cells and tissues.

Enzyme-mediated crosslinking reaction [57] can be considered as an alternative approach of in-situ chemical crosslinking reactions. Gao et al. (2014) [58] developed the enzyme-regulated protein hydrogels showing self-healing via dynamic covalent interaction between the glutaraldehyde and lysine residues of BSA and the two enzymes (glucose oxidase (GOX) and catalase (CAT)). The protein hydrogels including GOX and CAT showed 100% recovery, while the protein hydrogels without the two enzymes made 50% recovery. Xu et al. (2015) [59] first synthesized the hyaluronic acid–tyramine (HA–Tyr) conjugates. Then, HA–Tyr hydrogels were fabricated via the oxidative coupling reaction between the functional groups of tyramine of the HA–Tyr conjugates. Here, horseradish peroxidase (HRP) enzyme and hydrogen peroxide (H_2_O_2_) mediated the reaction. The human embryonic stem cells (hESCs) were incorporated and propagated in the HA–Tyr hydrogels. The compressive modulus of HA–Tyr hydrogels showing the best result of the proliferation of hESCs was about 350 pa, and also the differentiation of hESCs was possible in vivo and in vitro. The HA–Tyr hydrogels were considered as promising biomaterials for tissue regeneration.

## Therapeutic applications of injectable hydrogels

Since Wichterle and Lim (1960) [60] presented the first use of hydrogels as biomaterials for contact lenses that need oxygen permeability and compliance features, hydrogels have been applied to many different fields including pharmaceutical and medical industries. Even in the recent studies on the hydrogels to be used for the contact lenses, injectable hydrogels have been developing to deliver drugs to the eyes [61, 62]. Injectable hydrogels, which could be formed via in-situ gelation after injected in the desired site of the body, have begun to be used in therapeutic agent delivery systems as well as the tissue engineering because they are clinically more convenient and simple to be used than traditional hydrogels. The first application of injectable hydrogels for drug delivery and tissue engineering was carried out by Elisseeff et al. (1999) [63]. Their work was on that PEO hydrogel including the cells (or proteins) were formed from transdermally injected PEO dimethacrylate and chondrocytes (or albumins) under the irradiation of UV and visible lights. Their attempt happened approximately 30 years later since the sol-gel transition of PNIPAAM was reported by Heskins and Guillet (1968) [64].

For the use of chemotherapy, injectable hydrogels containing drugs that may produce the adverse effect on tissues when their release rate and amount are not controlled have regulated the drug loading and release by their degradation or swelling behavior [65–67]. Such control over the release of drugs has also importantly applied to other therapeutic agents to be used for regenerative medicine and immunotherapy. Like this, injectable hydrogels with more features compared to conventional hydrogels have been considered as excellent drug platforms giving better therapeutic efficacy. The recent studies of injectable hydrogels for drug delivery applications showed that multi-functional injectable hydrogels capable of entrapping and delivering the multiple therapeutic agents and other boosting substances such as MBP have been developing for the high therapeutic effect and the one-step treatment of various diseases and cancers [19–21, 34, 37]. Also, the studies on the “smart” injectable hydrogel drug delivery systems responded to external stimuli has been proceeding [33, 34, 38].

With regard to tissue engineering, the treatment of a bone (hard tissue) and soft tissue defect has been extensively studied for centuries, but the term “tissue engineering” has been used since the late 1980’s [68]. The initial attempt of cell seeding in tissues in the early 1970’s was followed by the experimental and clinical uses of collagen gel matrices for fibroblast cell seeding, and the development of scaffolds for cell delivery. Based on such performances, Langer and Vacanti (1993) [69] first published about tissue engineering, material candidates to be used as scaffolds, and their requirements and clinical cases applied in cartilage, blood vessel, nerve, cornea, skin, bone, several organs including liver and kidney, dental, and so on. Since then, a variety of studies of injectable hydrogels to be used in tissue engineering have been actively conducted for producing better repair effect. As like the studies on the injectable hydrogels for drug delivery, recently, multi-functional and hybrid as well as stimuli-responsive injectable hydrogels, are developing for tissue repair and regeneration [35, 40, 41, 58].

As seen in the summary of the applications of the injectable hydrogel systems reviewed in this article and the used therapeutic agents (see Table 1), injectable hydrogels have been widely researched for the use as carriers or scaffolds of therapeutic agents such as drugs, cells, proteins, and bioactive molecules (e.g., enzyme). As carriers, they have incorporated the agents and delivered them to the desired site in the body for the treatments of infectious and inflammatory diseases (Parkinson’s disease, bacterial and antimicrobial infection, and diabetes) and cancers (colon, lung, breast, ovarian, and lymphoma cancer). Next, as scaffolds, they have provided a flexible dwelling space of cells and stimuli to be immobilized and protected for the tissue repair and regeneration of the desired tissues (cartilage, bone, retina, brain, and, neural tissue repair, vascular regeneration, and wound healing). Moreover, they have been used for immunotherapy to enhance the immune system to prolong survival time by delivering the immunomodulatory agents. Table 1 also shows the injectable hydrogel systems, which are reviewed in this article, composed of natural, synthetic, and hybrid polymers and their crosslinking reactions/interactions and external conditions inducing gel formation. Additionally, their notable features and the list of references are seen in the table as well. Finally, the abbreviations used in the table are described in alphabetical order in footnotes below the table.

**Table 1:** The injectable hydrogel systems and their crosslinking reactions/interactions and applications

Injectable Hydrogel Systems	Crosslinking Reactions/Interactions	Applications (therapeutic agents)	Notes	References
Agar				
Agar/MBP	PC, HB/	Delivery of TD (DOX), chemotherapy for colon cancer, photothermal treatment by MBP	Temperature-sensitive release, temperature changed by MBP (a photoabsorbent)	[37]
Alginate				
Alginate-Ca	PC, EI/	Delivery of TD (MX) and TPs (SDF-1α and pDNA), breast cancer therapy	Self-healing, the release and therapeutic efficacy regulated by pulsatile ultrasound	[38]
OAlg/CMCS with GMs-TH	CC, SBR/	Bacterial infection therapy	Antibacterial and biodegradable properties	[52]
Chitosan				
Chitosan/gelatin/polydopamine	PC, EI/ MR, SBR,	Parkinson’s disease therapy, TD delivery (MT)	Mechanical strength improved via EI	[20]
Chitosan/gelatin/NDs	PC, EI, HB, VDWF/HB, DDI, HP	Scaffold for TE (embedded cell: VEGF)	Thermosensitive gelation, mechanical features improved by NDs, sustained release	[30]
Chitosan-polyaniline/oxidized dextran	CC, SBR/	Delivery of TDs (Amoxicillin and ibuprofen)	pH- and electric field- responsive, antibacterial activity	[33]
CEC/OSA	CC, MR/	TAD	Self-assembling, self-healing	[49]
GC/poly(EO-co-Gly)-CHO	CC, SBR/	Scaffold for TE (embedded cell: chondrocyte), cartilage repair	Mechanical properties and morphology controlled by poly(EO-co-Gly)-CHO	[51]
Chondroitin Sulfate (ChS)				
Pluronic F127-ChS/PEG	CC, DACR/	Scaffold for TE (embedded cell: BMP-4), bone defect repair	Gelation of Pluronic F127-ChS at 37 ^o^C, dual crosslinked Pluronic F127-ChS/PEG hydrogels	[44]
Collagen				
Collagen/Au	PC, EI/	PTT, PDT, TAD (TMPyP), breast cancer therapy	Shear-thinning, self-healing, antitumor efficacy affected by light irradiation	[21]
Collagen	PC, EI/	Scaffold for TE (embedded cell: osteoblast (OB)), bone repair	Successful bone tissue regeneration by OB and LIPUS	[39]
Dextrin				
CD/PEG-phospholipid/Fe_3_O_4_	PC, HI/	Hyperthermia cancer therapy, chemotherapy for preventing breast cancer recurrence, delivery of TDs (PTX, DOX)	PTX and DOX Released by ACMF induced heat	[34]
Gelatin				
Gelatin MPs	PC, EI/	Scaffold for TE (embedded TPs: VEGF and BMP2)	Release controlled by hydrogel degradation induced by CLSPA collagenase	[40]
Gelatin-tetrazine fg/Gelatin- norbornene fg	CC, DACR/	Scaffold for TE	Cell-compatible and enzymatically degradable properties	[43]
Hyaluronic acid (HA)				
HA-CD/ HA-AD, and HA-CD-MA/HA-AD-MA	PC, HGI, CC/	Delivery of TP (EPC), vascular regeneration	Shear-thinning, self-healing	[28]
HA-HMDA-cytosine/HA-HMDA-guanosine	PC, HB/	Delivery of TP (BSA), scaffold for TE	pH-sensitive and self-healing, gelation only at pH 6-8	[32]
HAMC	PC, EI, HB/	Delivery of TPs (RSCs, NSCs), retina and brain tissue therapy, scaffold for TE	Biodegradable and light-sensitive, HAMC-CD44 interaction	[35]
HA-Heparin (HA-HP)	CC, PIC/	Delivery of TP (AFS), wound healing	Crosslinking by UV light	[56]
HA-Tyr	CC, EMCR, OCR/	Scaffold for TE (embedded cell: hESC)	Highest hESC proliferation at HA-Tyr hydrogels with modulus of 350 Pa	[59]
Peptide/protein				
P1	PC, PI, HP/	Delivery of TDs (vitamin B12 and vancomycin, an antibiotic)	Thixotropic behavior at physiological condition	[23]
^F^G_d_/Ag	PC, PI/	Antimicrobial treatment with Ag	Mechanical stability for six months, high antimicrobial activity	[24]
PA	PC, HI, HB, EI/	Chemotherapy, delivery of TD (DOX)	PA concentration-dependent release behavior	[25]
Peptide/catalase/glucose oxidase	PC, EI/	Diabetic therapy, delivery of TP (insulin)	pH- and glucose-sensitive, self-assembling	[31]
Multi-domain peptides (MDPs) nanofiber/liposome NPs	PS, HB, HI/	Delivery of TPs (EGF, PlGF-1, MCP-1), Scaffold for TE	Release regulated via the hydrogel degradation by enzyme-mediation and the physical gelation by self-assembly	[41]
Protein and Protein-GOX-CAT (PGT)	CC, EMCR/	Scaffold for TE	Reversible self-healing, enzyme (glucose) mediated crosslinking by dynamic covalent interaction, 100% recovery PGT	[58]
PCL				
PEG-PCL-PEG	PC, HI/	Chemotherapy including diabetic treatment, delivery of TP (insulin)	Sol-gel transition at 37 ^o^C, lower viscosity than Pluronic®, Fickian release	[26]
PEG				
Polyamine-PEG-polyamine	PC, CC, EI, HB, PI/	Delivery of TPs (insulin, BSA, glucose, avidin, neutravidin, or IL-12)	Self-assembling at 30 ^o^C	[19]
PLga-PEG-PLga -chol fg/ PLga-CD fg	PC, HGI/	Scaffold for TE	Self-healing, self-assembling, properties regulated by the ratio of CD/Chol, high storage modulus	[27]
PCLA-PEG-PCLA/heparin	PC, HI, CC, MR/EI	Delivery of TP (lysozyme), immunotherapy	Thermosensitive gelation at BT, sustained release	[29]
PEG- aryl thiol fg/PEG-maleimide fg (or heparin-maleimide fg)	CC, MR/RLI	Delivery of TPs (FGF-2, cytokines, and immunomodulatory agents)	Light-and reducing environment- sensitive release	[36]
PEG/TMV_Cys_s and PEG/wt-TMV	CC, MR and PC/	Scaffold for TE	TMV with multi-functionality, storage moduli improved by adding TMV_Cys_s. No effect of wt-TMV on storage moduli	[48]
PEG/PPLL	CC, SBR/	Delivery of TDs (ME and 5FC), colon cancer therapy	Degradation and release controlled by pHs	[53]
Polyoxamine				
Polyoxamine-maleimide fg/ Polyoxamine-furyl fg	PC, CC, HI, DACR/	Delivery of TP (bevacizumab, an antibody), colon, lung, and breast cancer therapy	Physical and chemical crosslinking, triphasic release controlled by two polyoxamines	[45]
PVA				
PVA-Tyr and PVA-Tyr-sericin-gelatin (PVA-SG)	CC, PIC/	Scaffold for TE (embedded TP: Schwann)	Crosslinking by visible light, PVA-Tyr: no cell adhesion, PVA-SG: cell adhesion	[55]
PNIPAAm				
PNIPAAm/CNC	PC, VDWF, HB/	Delivery of TD (MT), wound dressing	Volume transition at 36-39 ^o^C, Mechanical strength increased by CNC	[22]

*Abbreviations: ACMF* Alternating current magnetic field, *AD* Adamantine, *AFS* Amniotic fluid-derived stem, *BMP2* bone morphogenetic protein 2, *BMP-4* Bone morphogenetic protein 4, *BSA* Bovine serum albumin, *BT* Body temperature, *CAT* Catalase, *CC* Chemical crosslinking, *CD* Cyclodextrin, *CEC* N-Carboxyethyl chitosan, *Chol* Cholesterol, *CLSPA collagenase* Chromatographically purified collagenase, *CMCS* carboxymethyl chitosan, *CNC* Cellulose nanocrystal, *DACR* Diels Alder click reaction, *DDI* dipole-dipole interaction, *pDNA* plasmid DNA, *Dox* doxorubicin, *EGF* Epidermal growth factor, *EI* Electrostatic interaction, *EMCR* Enzyme-mediated crosslinking reaction, *EPC* Endothelial progenitor cell, *FGF-2* Fibroblast growth factor, *fg* functional group, *5FU* 5-fluorouracil, *GC* glycol chitosan, ^*F*^*G*_*d*_ 20-deoxy-20-fluoroguanosine, *GMs* Gelatin microspheres, *GOX* Glucose oxidase, *HA* Hyaluronic acid, *HAMC* Hyaluronic acid-methylcellulose, *HB* Hydrogen bonding, *hESCs* Human embryonic stem cells, *HGI* Host-gest interactions, *HI* Hydrophobic interaction, *HMDA* Hexamethylenediamine, *IL-12* Interleukin-12, *LIPUS* Low-intensity pulsed and ultrasound, *MA* Methyl acrylate, *MBP* MoS2/Bi2S3-PEG, *MCP-1* monocyte chemoattractant protein-1, *ME* Metformin, *MGH* Magnetic gellan gum hydrogel, *MPs* microparticles, *MR* Michael reaction, *MT* Metronidazole, *MX* Mitoxantrone, *NDs* Nanodiamonds, *NPs* Nanoparticles, *NSCs* Neural stem and progenitor cells, *OAlg* Oxidized alginate, *OCR* Oxidative coupling reaction, *OSA* Oxidized sodium alginate, *P1* boc-AUDA-Phe-COOH (*AUDA* 11-aminoundecanoic acid, *Phe* L-phenylalanine), *PA* Peptide amphiphile, *PC* Physical crosslinking, *PCL* Polycaprolactone, *PCLA* Poly-(*ε*-caprolactone-co-lactide), *PDT* Photodynamic therapy, *PEG* Polyethylene glycol, *PI* π-Interaction, *PIC* Photo-initiated crosslinking, *PLga* Poly(L-glutamic acid), *PlGF-1* Placental growth factor-1, *PNIPAAm* Poly(N-isopropylacrylamide), *PPLL* PEG-b-poly(L-lysine), *Poly(EO-co-Gly)-CHO* Poly(ethylene oxide-co-glycidol)-CHO, *PTT* Photothermal therapy, *PTX* Paclitaxel, *PVA* Poly(vinyl alcohol), *PVA-SG* PVA-tyramine-sericin-gelatin, *RLI* Receptor–ligand interaction, *RSCs* Retinal stem cells, *SBR* Schiff base reaction, *SDF-1α* stromal cell-derived factor 1α, *TAD* Therapeutic agent delivery, *TDs* Therapeutic drugs, *TE* Tissue engineering, *TH* Tetracycline hydrochloride, *TPs* Therapeutic proteins, *TMPyP* meso-tetra(N-methyl-4-pyridyl) porphine tetrachloride (a photosensitive drug), *TMV* Tobacco mosaic virus, *wt-TMV* Wildtype TMV, *TMV*_*Cys*_*s* Cysteine-containing TMV mutants, *Tyr* Tyramine, *VDWF* van der Waals force, and *VEGF* Vascular endothelial growth factors

## Conclusions

In this review article, it has covered a variety of injectable hydrogels recently studied for delivery of therapeutic agents such as drugs, cells, proteins, and bioactive molecules for disease and cancer therapy and tissue repair and regeneration. Injectable hydrogels have been classified into chemically and physically crosslinked systems via different crosslinking reaction mechanisms and external stimuli. Accordingly, here they have been reviewed in this category. Physically crosslinked hydrogels fabricated via non-covalent interactions have been spontaneously and relatively merely prepared without any additional reactive reagents, but slightly mechanically weak to maintain themselves with preserving from the ambient influences such as pHs and temperatures for a long time, when injected into the body. On the other hand, the chemical crosslinked injectable hydrogels synthesized through the various in-situ chemical reactions without any toxic crosslinker and condition have shown to possess relatively higher mechanical stability and properties due to the covalent bonds formed from the chemical reaction, but their responses to external stimuli are expected to be limited. Regardless of the crosslinking mechanism, for the desired applications, the network morphology and properties of all injectable hydrogels have been controlled by regulating multiple factors that are the compositions, inclusions, crosslinking reactions, external conditions, and so on. There have been many cases where one chemical reaction or one environmental condition causes gelation, for instance, Diels Alder “click” reaction or temperature increase produces the network crosslinks of hydrogels. However, recently the advanced injectable hydrogels have been fabricating via combining two or three chemical and physical reactions or stimuli (e.g., dual crosslinked hydrogels) and with adding multi-functionality to carry multiple therapeutic agents. In those cases, the mechanical stability and properties and physical properties of injectable hydrogels formed after injection have been naturally improved to produce high load and controlled release of therapeutic agents, which consequently brought forth significant therapeutic efficacy. Ultimately, simple one-step therapy using various agents delivered by multi-functional injectable hydrogels with desirable physical and mechanical properties is what we pursue in the future work. In conclusion, injectable hydrogels are believed to be successful and promising biomaterials as therapeutic agent carriers (or scaffolds) for disease and cancer therapy and tissue repair and regeneration. Further developments in injectable hydrogels possessing the essential characteristics required for a variety of applications are anticipated to be achieved through many future studies.
